# Genomic Selection for F_1_ Hybrid Breeding in Strawberry (*Fragaria* × *ananassa*)

**DOI:** 10.3389/fpls.2021.645111

**Published:** 2021-03-04

**Authors:** Eiji Yamamoto, Sono Kataoka, Kenta Shirasawa, Yuji Noguchi, Sachiko Isobe

**Affiliations:** ^1^Graduate School of Agriculture, Meiji University, Kawasaki, Japan; ^2^Institute of Vegetable and Floriculture Science, National Agriculture and Food Research Organization, Tsu, Japan; ^3^Department of Frontier Research and Development, Kazusa DNA Research Institute, Kisarazu, Japan

**Keywords:** strawberry, F_1_ hybrid breeding, genomic selection, fruit hardness, pericarp color

## Abstract

Cultivated strawberry is the most widely consumed fruit crop in the world, and therefore, many breeding programs are underway to improve its agronomic traits such as fruit quality. Strawberry cultivars were vegetatively propagated through runners and carried a high risk of infection with viruses and insects. To solve this problem, the development of F_1_ hybrid seeds has been proposed as an alternative breeding strategy in strawberry. In this study, we conducted a potential assessment of genomic selection (GS) in strawberry F_1_ hybrid breeding. A total of 105 inbred lines were developed as candidate parents of strawberry F_1_ hybrids. In addition, 275 parental combinations were randomly selected from the 105 inbred lines and crossed to develop test F_1_ hybrids for GS model training. These populations were phenotyped for petiole length, leaf area, Brix, fruit hardness, and pericarp color. Whole-genome shotgun sequencing of the 105 inbred lines detected 20,811 single nucleotide polymorphism sites that were provided for subsequent GS analyses. In a GS model construction, inclusion of dominant effects showed a slight advantage in GS accuracy. In the across population prediction analysis, GS models using the inbred lines showed predictability for the test F_1_ hybrids and vice versa, except for Brix. Finally, the GS models were used for phenotype prediction of 5,460 possible F_1_ hybrids from 105 inbred lines to select F_1_ hybrids with high fruit hardness or high pericarp color. These F_1_ hybrids were developed and phenotyped to evaluate the efficacy of the GS. As expected, F_1_ hybrids that were predicted to have high fruit hardness or high pericarp color expressed higher observed phenotypic values than the F_1_ hybrids that were selected for other objectives. Through the analyses in this study, we demonstrated that GS can be applied for strawberry F_1_ hybrid breeding.

## Introduction

Cultivated strawberry (*Fragaria* × *ananassa*) is an allo-octoploid (2*n* = 8*x* = 56) species that originated from an interspecific hybridization between *Fragaria virginiana* and *Fragaria chiloensis* (Darrow, 1966). The cultivated strawberry is the most widely cultivated fruit crop in the world and has an annual global production exceeding 9 million tons in 2017 (FAOSTAT^1^). Because of its economic importance, breeding programs for cultivated strawberries are underway to improve fruit quality, disease resistance, and yield performance (Honjo et al., 2011; Lerceteau-Köhler et al., 2012; Roach et al., 2016; Gezan et al., 2017). Cultivated strawberries are usually propagated vegetatively from runners. Therefore, development and distribution of a new strawberry cultivar have been performed by selecting individuals with desirable characteristics and vegetative propagation. However, seedling production through runners carries a risk of infection with viruses and insects. Therefore, farmers and breeders are dedicating substantial efforts to protect strawberry runners from viruses and insects. To solve these problems, the development of seed-propagated strawberry has been proposed as an alternative strawberry breeding method. More specifically, production of F_1_ hybrid seeds has begun in several countries (Bentvelsen et al., 1997; Rho et al., 2012; Mori et al., 2015). The use of F_1_ hybrid breeding has two major advantages over the traditional vegetatively propagated strawberry breeding. One is the risk mitigation for infection by viruses and insects because seed infection has not been reported in major diseases in strawberry. Second, in general, F_1_ hybrids express high yield and high robustness against various stresses, although the genetic mechanism is largely unknown (Duvick, 2001). Therefore, F_1_ hybrid breeding has the potential to be a new standard in strawberry breeding.

Genomic selection (GS) is now widely used for genetic improvement of quantitative traits (Meuwissen et al., 2001). In GS, breeding selections are performed based on genetic potential that has been estimated from genome-wide genotype data; thus, GS can reduce the costs and effort required for phenotypic observation in plant breeding (Crossa et al., 2017). In F_1_ hybrid breeding, the number of parental combinations that should be tested exponentially increases as the number of candidate parents (i.e., inbred lines) increases. Therefore, the pre-selection of promising F_1_ hybrids (or the pre-removal of non-promising F_1_ hybrids) by using GS considerably contributes to efficient F_1_ hybrid breeding (Xu et al., 2014; Acosta-Pech et al., 2017; Basnet et al., 2019). In GS, a training population, which has been phenotyped and genotyped, is used to construct a model that predicts the genetic potential of unphenotyped individuals using genome-wide genotype data. Therefore, the availability of genome-wide genotyping platforms is necessary to conduct GS. Recent advances in genome sequencing technologies have enabled the development of analytical platforms for complicated genomes such as allo-octoploid strawberry. The first genome sequence assembly of allo-octoploid strawberry was conducted for the cultivar ‘Reikou’ (Hirakawa et al., 2014). Recently, a chromosome-scale assembly was developed for the cultivar ‘Camarosa’ (Edger et al., 2019). These reference genomes enable the detection of large numbers of single nucleotide polymorphisms (SNPs) and subsequent genetic analyses. Bassil et al. (2015) developed a high-density SNP genotyping array that enabled the construction of genetic linkage maps in allo-octoploid strawberry (Nagano et al., 2017). The genetic linkage maps revealed that subgenome-specific loci were randomly located across the genomes. Hardigan et al. (2019) conducted genotyping-by-sequencing (GBS) for a diversity panel of *Fragaria* species, including *F. × ananassa, F. chiloensis*, and *F. virginiana*, which revealed macrosynteny between cultivated strawberries and wild progenitors. These genome-wide genotyping platforms for strawberry have also been applied for GS. Gezan et al. (2017) assessed the potential of GS to improve basic agronomic traits such as fruit quality and yield performance. Pincot et al. (2020) evaluated GS model accuracy for soil-borne disease resistance by using a genetically diverse population. However, to the best of our knowledge, no study has applied GS for F_1_ hybrid breeding in strawberry.

In this study, we conducted a potential assessment of GS in strawberry F_1_ hybrid breeding. For this objective, we used 105 inbred lines that were developed as candidate parents in an F_1_ hybrid-breeding program. In addition, we developed 275 test F_1_ hybrids whose parental combinations were randomly selected from the 105 inbred lines. The genotype and phenotype data for these populations were used to construct GS models for vegetative and fruit-related traits. Then, the GS models were used for phenotype prediction of 5,460 possible F_1_ hybrids from the 105 inbred lines. Finally, we conducted breeding selection for the characteristics of fruit hardness and pericarp color to demonstrate the efficacy of GS in strawberry F_1_ hybrid breeding.

## Materials and Methods

### Plant Materials

**FIGURE 1:**
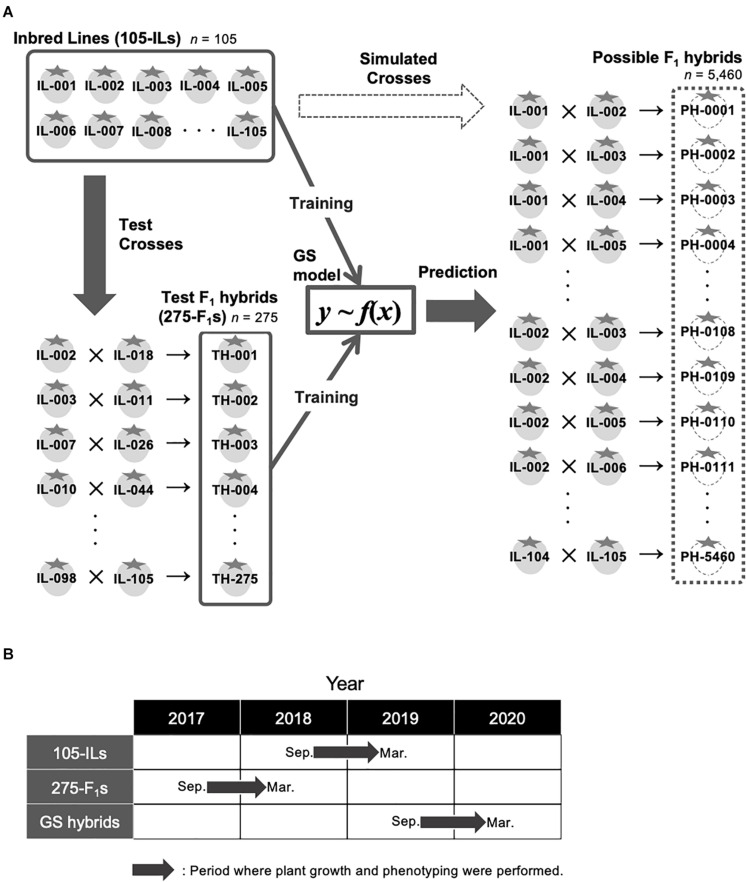
Experimental design in this study. **(A)** Schematic representation of GS strategy in this study. **(B)** Periods where plant growth and phenotyping were conducted in this study. ‘105-ILs’ indicates the 105 inbred lines. ‘275-F_1_s’ indicates the 275 test F_1_ hybrids. ‘GS hybrids’ indicates the F_1_ hybrids that were selected for the pilot GS experiment in this study.

**FIGURE 2:**
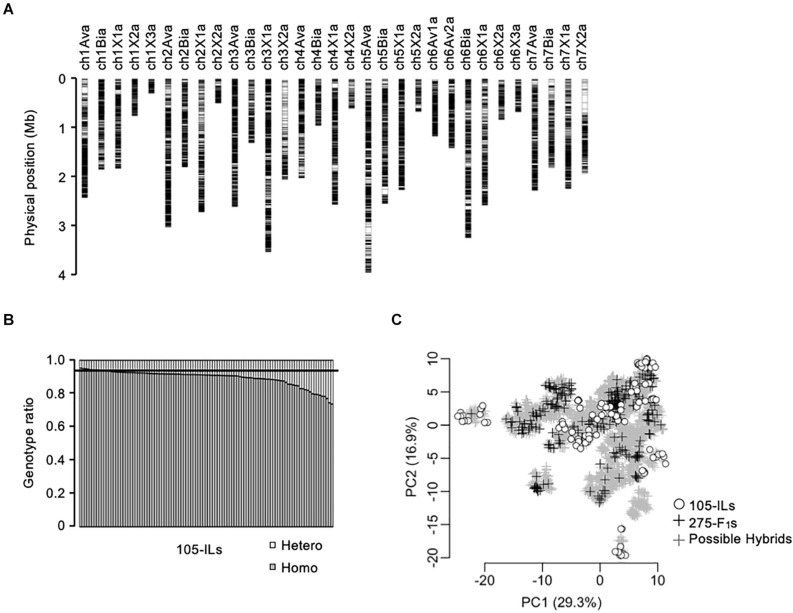
Genetic relationship of the populations in this study. **(A)** Chromosomal distribution of 28,011-SNPs used in this study. **(B)** Homozygosity of the 105 inbred lines. The horizontal line indicates expected homozygosity genotype ratio (i.e., 0.9375). **(C)** Principal component analysis of the populations in this study based on 28,011-SNP genotypes. The gray crosses indicate the 5,460 possible F_1_ hybrids. The black crosses indicate the 275 test F_1_ hybrids. The white circles indicate the 105 inbred lines.

A total of 105 inbred lines were developed for candidate parents of hybrid breeding at the Institute of Vegetable and Floriculture Science, National Agriculture and Food Research Organization, Ano, Tsu, Japan (Figure 1A). The ancestors of the 105 inbred lines are strawberry cultivars that include ‘Aiberry,’ ‘Aistro,’ ‘Akanekko,’ ‘Akihime,’ ‘Amaou,’ ‘Asukaruby,’ ‘Athene,’ ‘Benihoppe,’ ‘Chukan-bohon Nou 2,’ ‘Hinomine,’ ‘Houkou wase,’ ‘Kentaro,’ ‘Kurume 58,’ ‘Pajaro,’ ‘Sachinoka,’ ‘Sagahonoka,’ ‘Santigo,’ ‘Satsumaotome,’ ‘Tochihime,’ ‘Tochinomine,’ ‘Toyonoka,’ and ‘Yumeamaka.’ After three cycles of recurrent random crosses, 105 progenies that did not show low fertility and aberrant morphology were selected. Thus, genomes of the progenies consist of a mixture of ancestor genomes. Four generations of single seed descent were then performed to develop the inbred lines. In addition, 275 test F_1_ hybrids were developed for GS model training (Figure 1A). Parental combinations of the test 275 F_1_ hybrids were randomly selected from the 5,460 possible combinations (i.e., we did not consider whether a line was used for seed- or pollen-parent). Validity of the selected parental combinations as a GS training population was confirmed using the genetic relationship between the 275 test F_1_ hybrids and the other possible parental combinations (see below; Figure 2C). Figure 1A represents the GS breeding scheme in this study and the role of the population.

### Phenotyping

Strawberry agronomic traits analyzed in this study are summarized in Table 1. Each phenotypic value in this study was the average of six plants per genotype. Leaf area was determined by approximating the ellipse, calculated as leaf length (cm) × leaf width (cm) × 3.14. Brix values were measured using a refractometer, PAL-1 (ATAGO, Tokyo, Japan). Fruit hardness was measured using a digital force gage DS2-5N, and the data were analyzed using the ZP-Recorder software (IMADA, Aichi, Japan). To measure fruit hardness, fruits were compressed using a 2 rigid plunger at a 10 mm/min compression speed. To evaluate pericarp color, lightness (L^∗^) and hue (a^∗^, b^∗^) were measured using a colorimeter, CR-20 (KONICA MINOLTA, Tokyo, Japan). Pericarp color value was calculated as L^∗^ × b^∗^/a^∗^, where lightness (L^∗^) and hue (a^∗^, b^∗^) were measured using the colorimeter. The distribution of the phenotypic values are shown in Supplementary Figure 1. Figure 1B represents periods where plant growth and phenotyping for each population were conducted. Plant growth and phenotyping were performed using elevated cultivation system in a greenhouse at the Institute of Vegetable and Floriculture Science, National Agriculture and Food Research Organization, Ano, Tsu, Japan.

**TABLE 1:** Strawberry traits and their estimated heritability.

Trait	Details	Estimated heritability^*a*^		
		105-ILs	275-F_1_s	
		*h*^2^_*A*_	*h*^2^_*A*_	*h*^2^_*AD*_
Petiole length	Petiole length (cm)	0.630	0.715	0.733
Leaf area	Ellipse approximation calculated as leaf length (cm) × leaf width (cm) × 3.14	0.589	0.769	0.78
Brix	Degree of brix, measured using a refractometer	0.357	0.724	0.765
Fruit hardness	Samples compressed with a 2φ rigid plunger and a compression speed of 10 mm/min	0.400	0.618	0.691
Pericarp color	Calculated as L* × b*/a*, where lightness (L*) and hue (a*, b*) were measured by a colorimeter	0.260	0.739	0.783

*^a^105-ILs: 105 inbred lines; 275-F_1_s: 275 test F_1_ hybrids; h^2^_A_: heritability based on additive effect model; h^2^_AD_: heritability based on additive plus dominant effect model.*

### Whole Genome Shotgun (WGS) Sequencing

Genomic DNA of the 105 inbred lines was extracted from the leaves using the Qiagen DNeasy Plant Mini Kit (Qiagen, Hilden, Germany). The DNA was physically sheared into approximately 350bp fragments using Covaris S220 (Covaris, Woburn, MA, United States) and fragments of 300–400 bp in length were fractionated with the Sage Science BluePippin (Sage Science, Inc., Beverley, MA, United States). The fractionated DNA was used for DNA library construction with Illumina TruSeq DNA PCR-Free Library Preparation Kit (Illumina, San Diego, CA, United States) in accordance with the manufacturer’s protocols. Sequencing of the DNA library was conducted with Illumina HiSeq X with 2 × 150 paired end reads. The obtained reads were subjected to quality control as follows. Bases with quality scores less than 10 were filtered using PRINSEQ version 0.20.4 (Schmieder and Edwards, 2011) and adaptor sequences in the reads were trimmed using fastx_clipper from the FASTX-Toolkit version 0.0.13^2^. The filtered reads were mapped onto the reference sequence of the strawberry genome (FAN_r2.3; Strawberry GARDEN^3^) using Bowtie 2 version 2.3.2 (Langmead and Salzberg, 2012) with parameters of maximum fragment size length 1000 (X = 1000), in the ‘–sensitive’ preset of the ‘–end-to-end’ mode. The resulting binary alignment map (BAM) files were subjected to variant calling using the mpileup option (parameters of -Duf) of SAMtools version 0.1.20 and the view option (parameters of -vcg) of BCFtools (Li et al., 2009). The variants were filtered using VCFtools version 0.1.13 (Danecek et al., 2011) with the following parameters: minimum read depth = 10 (–minDP 10), minimum mean read depth = 1000 (–min-meanDP 1000), maximum mean read depth = 6000 (–max-meanDP 6000), minimum minor allele frequency = 0.05 (–maf 0.05), minimum genotype quality = 20 (–minGQ 20), and maximum proportion of missing data = 0.1 (–max-missing 0.1). Linkage disequilibrium (LD)-based variant pruning was performed using BCFtools (Li et al., 2009) with parameters of maximum LD = 0.95 (−l 0.95), and window size = 1000 bp (-w 1000). The missing genotypes were estimated using the R package missForest version 1.4 with default parameter settings (Stekhoven and Bühlmann, 2012). The SNPs whose frequency of heterozygotes was greater than 0.25 were filtered out because these may not be subgenome-specific loci (Supplementary Figure 2). Finally, the SNPs detected on unanchored scaffolds of the reference genome were excluded from further analyses because of ambiguity in their chromosomal location. To investigate the genetic relationship between the populations in this study, we conducted a principal component analysis (PCA) with the R function ‘prcomp.’

### Heritability

In this study, we estimated trait heritability using two methods. One is the additive effect heritability (${\hat{h}}_{A}^{2}$), which was calculated using the following equation:

(1) $${\hat{h}}_{A}^{2}={\hat{\sigma }}_{A}^{2}/\left({\hat{\sigma }}_{A}^{2}+{\hat{\sigma }}_{e}^{2}\right)$$

where ${\hat{\sigma }}_{A}^{2}$ and ${\hat{\sigma }}_{e}^{2}$ are additive genetic variance and error variance, respectively. The second heritability is additive plus dominant effect heritability (${\hat{h}}_{A}^{2}$), which was calculated as the following:

(2) $${\hat{h}}_{AD}^{2}=\left({\hat{\sigma }}_{A}^{2}+{\hat{\sigma }}_{D}^{2}\right)/\left({\hat{\sigma }}_{A}^{2}+{\hat{\sigma }}_{D}^{2}+{\hat{\sigma }}_{e}^{2}\right),$$

where ${\hat{\sigma }}_{D}^{2}$ is the dominant genetic variance. ${\hat{\sigma }}_{A}^{2}$ and ${\hat{\sigma }}_{D}^{2}$ were estimated using the additive (**A**) and dominance (**D**) relationship matrices, respectively (Endelman and Jannink, 2012; Vitezica et al., 2013). For ${\hat{h}}_{A}^{2}$, ${\hat{\sigma }}_{A}^{2}$was estimated using the following:

(3) $$\hat{\text{V}}=\text{A}{\hat{\sigma }}_{A}^{2}+\text{I}{\hat{\sigma }}_{e}^{2},$$

where **I** is an *n* × *n* identity matrix, and $\hat{V}$ is the phenotypic variance–covariance matrix. For ${\hat{h}}_{AD}^{2}$, ${\hat{\sigma }}_{A}^{2}$ and ${\hat{\sigma }}_{D}^{2}$ was estimated by fitting:

(4) $$\hat{\text{V}}=\text{A}{\hat{\sigma }}_{A}^{2}+\text{D}{\hat{\sigma }}_{D}^{2}+\text{I}{\hat{\sigma }}_{e}^{2}$$

Fitting of eq. 3 and 4 were performed using the function ‘BGLR’ in the R package BGLR version 105 (Pérez and de Los Campos, 2014). In the estimation of ${\hat{h}}_{AD}^{2}$, we did not show the value of each variance component (i.e., ${\hat{\sigma }}_{A}^{2}$ and ${\hat{\sigma }}_{D}^{2}$) since precise calculation of each variance component was difficult because of the correlation between **A** and **D**.

### GS Model Construction

In this study, we tested five statistical models to construct the GS models. The genomic best linear unbiased prediction (GBLUP; VanRaden, 2008), Bayes B (BB; Meuwissen et al., 2001), and Bayesian Lasso (BL; Park and Casella, 2008) are linear models. For the linear models, we compared two options: additive effect model and additive plus dominant effect model. As for nonlinear models, we tested reproducing kernel Hilbert spaces regression (RKHS; Gianola and Van Kaam, 2008) and random forest (RF; Breiman, 2001). We did not test the additive plus dominant effect model for the two nonlinear models because these models have been designed to incorporate dominant effects.

The additive and additive plus dominant effect models for GBLUP were equivalent to eq. 3 and 4. Fitting of the GBLUP models were performed using the function ‘BGLR’ in the R package BGLR version 1.0.5 (Pérez and de Los Campos, 2014). The additive plus dominant effect model for BB and BL were designed using the concatenation of genotype matrix and heterozygosity matrix as the explanatory variables. The genotype matrix includes SNP genotype values that were coded as {−1, 0, 1} = {aa, Aa, AA}. In the heterozygosity matrix, heterozygosity of the SNP genotype was coded as {0, 1, 0} = {aa, Aa, AA}. BB and BL in this study were performed using the function ‘vigor’ in the R package VIGoR version 1.0 (Onogi and Iwata, 2016). RKHS was performed using function ‘kin.blup’ in the R package rrBLUP version 4.6.1 (Endelman, 2011). RF was performed using function ‘randomForest’ in the R package randomForest version 4.6 (Liaw and Wiener, 2002).

### Cross-Validation

Two-fold cross-validation was performed to evaluate the accuracy of the GS models in this study. We performed 50 replicates for each trait and the same fold was used between combinations of statistical models and traits. The predictive accuracy was measured as the Pearson’s correlation coefficient between the predicted and observed phenotypic values using the R function ‘cor.test.’

### Genotypes of the Possible F_1_ Hybrids

The SNP genotypes of the 5,460 possible F_1_ hybrids (Figure 1A) were determined using the following rules. If the genotype of an SNP in parent 1 (P1) and parent 2 (P2) was {P1, P2} = {AA, AA}, then the genotype in the F_1_ hybrid was {F_1_} = {AA}. In a similar manner, {P1, P2, F_1_} = {aa, aa, aa} and {P1, P2, F_1_} = {AA, aa, Aa}. If the genotype of the parent(s) was heterozygous, then we determined the genotype of the F_1_ hybrids as {P1, P2, F_1_} = {Aa, aa, Aa}, {P1, P2, F_1_} = {Aa, Aa, Aa}, and {P1, P2, F_1_} = {aa, Aa, Aa}.

## Results

### Genetic Relationship Between Parental Inbred Lines and the F_1_ Hybrids

A total of 28,011-SNP sites were detected by WGS for the 105 inbred lines (Figure 2A). The selected SNPs covered the entire subgenomic regions of the reference genome (FAN_r2.3; Strawberry GARDEN^3^). Because the SNPs were almost evenly distributed on the subgenomes, we evaluated the homozygosity of the 105 inbred lines as the ratio of homozygous SNP genotypes of each line (Figure 2B). The expected homozygosity of the 105 inbred lines (i.e., after four generations of selfings) was 0.9375. However, 98 out of 105 inbred lines were below this expected homozygosity ratio (Figure 2B).

The genetic relationship between the training population and breeding population is an important factor for the success of GS. In this study, 105 inbred lines and 275 test F_1_ hybrids were provided as training populations, while 5,460 possible F_1_ hybrids were the test population (Figure 1A). It should be noted that the 5,460 possible F_1_ hybrids include the 275 test F_1_ hybrids. The genetic relationship was investigated using principal component analysis (Figure 2C). The first and second principal components represented 29.3% and 16.9% of the total genetic variations, respectively. The distribution of possible F_1_ hybrids indicated that some hybrids were genetically close to the 105 inbred lines, but most of the hybrids were genetically distinct from the inbred lines (Figure 2C). Contrarily, the 275 test F_1_ hybrids showed more genetic diversity, and most of the possible hybrids showed a close genetic relationship with some of the 275 test F_1_ hybrids (Figure 2C). This result indicates that the 275 test F_1_ hybrids are genetically suitable for constructing GS models for predicting phenotypes of all possible F_1_ hybrids.

### Trait Heritability

We estimated trait heritability for both the 105 inbred lines and the 275 test F_1_ hybrids (Table 1). The value of heritability was high for all traits, indicating a significant contribution of genetic effects on the phenotypic values, and, thus, the applicability of GS for these traits. The higher heritability observed in the 275 test F_1_ hybrids than that of the 105 inbred lines may be attributable to larger genetic variation in the test F_1_ hybrids (Figure 2C). In the F_1_ hybrids, the inclusion of the dominant effect increased estimated heritability in all analyzed traits in this study (Table 1). This result suggests that dominant effects make a notable contribution to phenotypic values.

The inbred lines showed more heterozygosity than the that of the theoretically expected values (Figure 2B); thus, we estimated the heritability of the inbred lines by using a dominance relationship matrix. Unexpectedly, the use of the dominance relationship matrix resulted in extremely high heritability values (Supplementary Table 1). Although it is possible that the heterozygous regions in the inbred lines have significant effects on phenotypic values, it seems that the high heritability values are due to accidental overfitting as the dominant effects have little impact on GS accuracy (see below, Table 2).

**TABLE 2:** Accuracy of GS models in traits analyzed in this study.

Population^*a*^	Trait	Model^*b*^							
		GBLUP-A	GBLUP-AD	BB-A	BB-AD	BL-A	BL-AD	RKHS	RF
105-ILs	Petiole length	0.771 (0.036)	0.773 (0.035)	0.764 (0.034)	0.764 (0.034)	0.769 (0.038)	0.773 (0.037)	0.773 (0.036)	0.744 (0.047)
	Leaf area	0.590 (0.054)	0.611 (0.049)	0.588 (0.043)	0.600 (0.043)	0.580 (0.060)	0.597 (0.055)	0.587 (0.057)	0.575 (0.054)
	Brix	0.279 (0.091)	0.288 (0.089)	0.258 (0.095)	0.270 (0.094)	0.294 (0.082)	0.306 (0.081)	0.375 (0.073)	0.309 (0.087)
	Fruit hardness	0.514 (0.063)	0.520 (0.063)	0.526 (0.059)	0.528 (0.059)	0.466 (0.073)	0.474 (0.073)	0.505 (0.076)	0.498 (0.074)
	Pericarp color	0.306 (0.073)	0.310 (0.074)	0.316 (0.070)	0.317 (0.070)	0.260 (0.087)	0.265 (0.086)	0.286 (0.097)	0.291 (0.085)
275-F_1_s	Petiole length	0.584 (0.043)	0.585 (0.044)	0.559 (0.043)	0.564 (0.043)	0.608 (0.048)	0.593 (0.052)	0.568 (0.048)	0.574 (0.049)
	Leaf area	0.672 (0.032)	0.684 (0.034)	0.644 (0.029)	0.659 (0.030)	0.686 (0.039)	0.683 (0.040)	0.675 (0.036)	0.676 (0.041)
	Brix	0.565 (0.049)	0.564 (0.052)	0.568 (0.044)	0.566 (0.046)	0.532 (0.053)	0.526 (0.055)	0.553 (0.056)	0.535 (0.053)
	Fruit hardness	0.712 (0.036)	0.703 (0.035)	0.702 (0.035)	0.699 (0.035)	0.702 (0.039)	0.688 (0.038)	0.694 (0.037)	0.661 (0.039)
	Pericarp color	0.667 (0.038)	0.668 (0.037)	0.644 (0.035)	0.642 (0.034)	0.662 (0.042)	0.659 (0.041)	0.668 (0.037)	0.658 (0.042)

*Accuracy was evaluated as a Pearson’s correlation coefficient between phenotypic and predicted values from 50-cycles of 2-fold cross-validation. The values in parentheses are standard deviations.^a^105-ILs, 105 inbred lines; 275-F_1_s, 275 test F_1_ hybrids.^b^GBLUP, Genomic best linear unbiased prediction; BB, Bayes B; BL, Bayesian Lasso; RKHS, Reproducing kernel Hilbert space regression; RF, Random forest.; -A, additive effect model; -AD, additive plus dominant effect model.*

### GS Model Accuracy

In this study, we have two populations that can be provided as a training population for GS model construction (i.e., the 105 inbred lines and the 275 test F_1_ hybrids). To assess the validity of GS for strawberry population in this study, we evaluated GS model accuracy using three methods. The first and second methods were within population cross-validation for the 105 inbred lines and the 275 test F_1_ hybrids, respectively. The third method was across population prediction that predicted phenotypes of the 275 test F_1_ hybrids by using the 105 inbred lines as a training population and vice versa.

#### Cross-Validation Within the 105 Inbred Lines

Petiole length exhibited the highest accuracy while Brix and pericarp color showed the lowest accuracy (Table 2). These GS accuracies were correlated with the estimated heritabilities (Table 1). The difference in the GS model construction methods showed little difference in accuracy (Table 2). However, in linear models, additive plus dominant effect models showed higher accuracy than the additive effect model in all cases, although these were not statistically significant (Table 2). These results suggested that the heterozygous regions remained in the 105 inbred lines (Figure 2B), and the dominant effects contributed to the phenotypic values.

#### Cross-Validation Within the 275 Test F_1_ Hybrids

In fruit quality related traits that consisted of Brix, fruit hardness, and pericarp color, GS model accuracy was higher in the 275 test F_1_ hybrids than in the 105 inbred lines (Table 2). This result could be attributed to higher heritability in the 275 test F_1_ hybrids (Table 1). Unlike the results for the 105 inbred lines, the advantage of additive plus dominant effects in linear methods was not clear in the 275 test hybrids (Table 2). As in the case of the 105 inbred lines, the difference in GS model construction methods showed little difference in accuracy (Table 2).

#### Across Population Prediction

**FIGURE 3:**
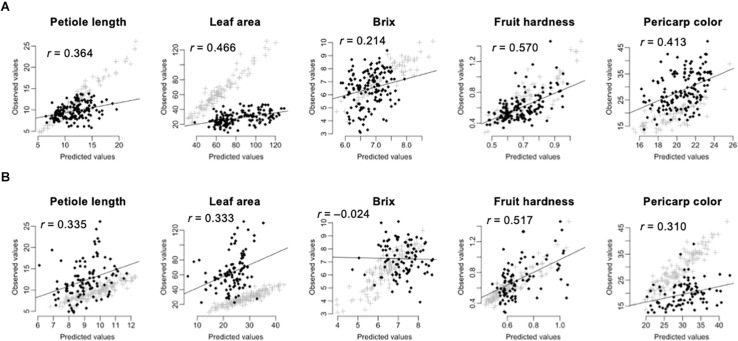
Across population prediction accuracy of GS models. The predicted values were calculated by GBLUP with additive plus dominant effect model. The value in each panel indicates the correlation coefficient (*r*) between predicted and observed values. The black squares indicate the predicted values. The gray crosses indicate the fitted values in the training population. **(A)** Prediction of the 275 test F_1_ hybrids by using the 105 inbred lines as the training population. **(B)** Prediction of the 105 inbred lines by using the 275 test F_1_ hybrids as the training population.

Prediction of the 275 test F_1_ hybrids using the 105 inbred lines showed higher accuracy than prediction of the 105 inbred lines using the 275 test F_1_ hybrids (Table 3 and Figure 3). In linear models, additive plus dominant effect models showed higher accuracy than additive effect models except for pericarp color in the prediction of the 275 test F_1_ hybrids (Table 3). This result indicated the contribution of dominant effects to the strawberry phenotypes. In both cases, the accuracy for Brix was lower than that for the other traits (Table 3). In particular, GS models showed no predictability in the 105 inbred lines (Table 3 and Figure 3). These results indicate difficulty in the application of GS for Brix in this study. Because both the 105 inbred lines and the 275 test F_1_ hybrids showed GS predictability except for Brix (Figure 3), we concluded that both populations can be used in the GS model construction for F_1_ hybrid breeding in strawberry.

**TABLE 3:** Across population prediction accuracy of GS models.

Training population^*a*^	Test population	Trait	Model^*b*^							
			GBLUP-A	GBLUP-AD	BB-A	BB-AD	BL-A	BL-AD	RKHS	RF
105-ILs	275-F_1_s	Petiole length	0.352	0.364	0.326	0.338	0.369	0.375	0.359	0.343
		Leaf area	0.440	0.466	0.383	0.415	0.461	0.481	0.442	0.463
		Brix	0.167	0.214	0.087	0.148	0.268	0.284	0.241	0.254
		Fruit hardness	0.557	0.570	0.588	0.591	0.454	0.455	0.582	0.487
		Pericarp color	0.430	0.413	0.438	0.425	0.412	0.403	0.435	0.400
275-F_1_s	105-ILs	Petiole length	0.309	0.335	0.330	0.350	0.297	0.336	0.338	0.355
		Leaf area	0.282	0.333	0.296	0.364	0.261	0.318	0.317	0.402
		Brix	−0.049	−0.024	−0.061	−0.036	−0.043	0.013	−0.029	0.021
		Fruit hardness	0.497	0.517	0.522	0.543	0.482	0.501	0.499	0.467
		Pericarp color	0.302	0.310	0.341	0.344	0.279	0.287	0.299	0.316

*The accuracy was evaluated as a Pearson’s correlation coefficient between phenotypic and predicted values.^a^105-ILs, 105 inbred lines; 275-F_1_s, 275 test F_1_ hybrids.^b^GBLUP, Genomic best linear unbiased prediction; BB, Bayes B; BL, Bayesian Lasso; RKHS, Reproducing kernel Hilbert space regression; RF, Random forest.; -A, additive effect model; -AD, additive plus dominant effect model.*

### GS in Strawberry F_1_ Hybrid Breeding

Because both the 105 inbred lines and the 275 test F_1_ hybrids showed GS predictability (Figure 3), we reconstructed GS models using both populations as the training populations. The GS model was constructed using GBLUP with additive plus dominant effects. GBLUP was selected because (1) the difference in GS model construction methods showed little difference in accuracy (Tables 2, 3), and (2) GBLUP is one of the most widely used methods for GS model construction (VanRaden, 2008). As an option of GBLUP, an additive plus dominant effects model was used because inclusion of dominant effects increased GS accuracy in most cases in this study (Tables 2, 3). The GS models were then used for phenotype prediction of 5,460 possible F_1_ hybrids (Figure 1A). Comparison of GS predicted values between F_1_ hybrids and their parents indicated that the GS predicted values in the F_1_s were not simply intermediate of their parents (Supplementary Figure 3). This comparison also indicated that dominant effects contributed the GS prediction, despite the fact that the dominant effects had little impact on overall GS accuracy (Table 2).

**FIGURE 4:**
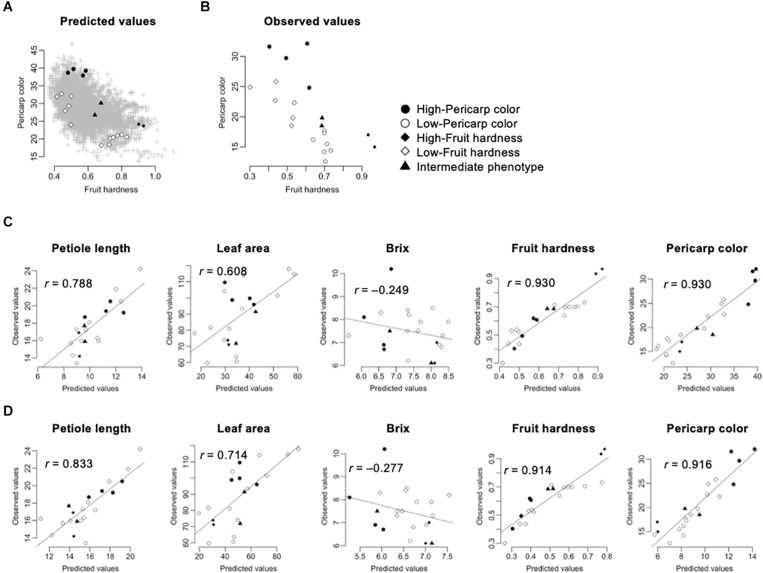
Application of GS for strawberry hybrid breeding. The predicted values were calculated by GBLUP with additive plus dominant effect model. The gray crosses indicate the 5,460 possible F_1_ hybrids. The black and white circles indicate the F_1_ hybrids selected for high- and low-pericarp color, respectively. The black and white squares indicate the F_1_ hybrids selected for high- and low-fruit hardness, respectively. The black triangles indicate the F_1_ hybrids selected for intermediate phenotypes. **(A)** Distribution of predicted fruit hardness and pericarp color in 5,460 possible F_1_ hybrids. **(B)** Distribution of observed phenotypic values in the selected F_1_ hybrids. **(C)** Accuracy of GS models in the selected F_1_ hybrids. The value in each panel indicates the correlation coefficient (*r*) between predicted and observed values. The GS models were constructed using both the 105 inbred lines and the 275 test F_1_ hybrids as the training populations. **(D)** Accuracy of GS models in the selected F_1_ hybrids. The value in each panel indicates the correlation coefficient (*r*) between predicted and observed values. The GS models were constructed using the 105 inbred lines as the training population.

In this pilot experiment, we focused on the fruit hardness and pericarp color. These traits were selected because of their agronomic importance and high accuracy in GS models (Tables 2, 3). We selected five classes of F_1_s, whose performances were predicted using the GS models: (1) high-pericarp color, (2) low-pericarp color, (3) high-fruit hardness, (4) low-fruit hardness, and (5) intermediate phenotype. Figure 4A shows the distribution of GS predicted values for 5,460 possible F_1_ hybrids and the F_1_ hybrids from the five classes. We then developed F_1_ hybrids from the five classes and performed phenotyping (Figure 1B). Parental combinations of the top or bottom predicted values were not selected because these crossings were difficult due to gaps in flowering time. Figure 4B shows the distribution of the observed phenotypic values in the F_1_ hybrids from the five classes. The distribution of phenotypic values of the F_1_ hybrids corresponded with the GS predicted values (Figures 4A,B). Figure 4C shows the relationship between GS predicted and observed phenotypic values in the F_1_ hybrids from the five selected classes. It should be noted that the extremely high correlation in fruit hardness and pericarp color is attributable to the selection of parental combinations (i.e., F_1_ hybrids with high and low were selected for these traits; Figure 4A). Interestingly, GS predicted values were correlated with observed phenotypic values in petiole length and leaf area (Figure 4C). These results reconfirmed that the GS models can predict the phenotype of F_1_ hybrids. However, the GS models did not show predictability in Brix (Figure 4C). This result is reasonable because the results from across population prediction accuracy suggested difficulty in GS prediction of this trait (Table 3 and Figure 3).

Because GS models based on the 105 inbred lines showed predictability for phenotypic value of the 275 test F_1_ hybrids (Figure 3), we investigated whether similar results could be obtained in the hybrids selected for the GS. Figure 4D represents the correlation between the observed phenotypic values and predicted values from GS models using the 105 inbred lines as the training population. The correlation coefficients were equivalent between GS models using just the 105 inbred lines and GS models using both the 105 inbred lines and the 275 test F_1_ hybrids (Figures 4C,D), as in the results from cross-validation based on across population prediction (Figure 3B). These results reconfirmed the conclusions drawn from the cross-validation (Figure 3B); namely, data from the parental inbred lines were sufficient in predicting the phenotypes of strawberry F_1_ hybrids in this study if estimated GS accuracy was sufficient in cross-validation.

## Discussion

F_1_ hybrid breeding in strawberry is a promising strategy to avoid the risk of infection with viruses and insects in traditional runner-based cultivars (Bentvelsen et al., 1997; Rho et al., 2012; Mori et al., 2015). In this study, we conducted a potential assessment of GS for strawberry F_1_ hybrid breeding (Tables 2, 3 and Figure 3) and demonstrated its efficacy in a practical breeding selection experiment (Figure 4).

The WGS in this study detected 28,011-SNP sites covering the entire subgenomic regions of the reference genome (Figure 2A; Strawberry GARDEN^3^). This result was consistent with a previous study that indicated the random location of subgenome-specific loci on the entire genome (Nagano et al., 2017). Diploid-like pairing and availability of genotype data covering entire subgenome regions enables direct application of genetic analysis methods designed for diploid to allo-polyploids. In this study, we did not use SNPs that are not from subgenome-specific loci. In genotype calling using next-generation sequencing data, abundant sequence data are needed for SNPs with higher ploidy levels (Gerard et al., 2018). In addition, sufficient prior information such as genotype of population in a simple pedigree is necessary, especially in allo-polyploids (Clark et al., 2019). Preparation of both data is difficult in strawberry genetic study, and therefore, subgenome-specific loci were used for GS in this study and the previous studies (Gezan et al., 2017; Pincot et al., 2020). Recent advances not only in throughput of sequencers but also experimental methods are increasing accuracy of genotype calling for SNPs with higher ploidy levels (Wadl et al., 2018). Application of the latest technologies will enable precise genotype calling for polyploid SNPs and valid application of the SNPs in GS.

When we investigated the homozygosity of the 105 inbred lines, 98 of 105 inbred lines were below the expected homozygosity ratio (Figure 2B). It is possible that this result was due to SNP genotypes that were not from subgenome-specific loci. However, we believe that the contribution of this possibility was not significant because we had removed SNP sites whose frequency of heterozygotes was greater than 0.25 (see section “Materials and Methods”). This filtering resulted in an increase, rather than a decrease, in the observed homozygosity, as there were numerous SNP sites whose frequency of heterozygotes was greater than 0.25 (Supplementary Figure 2). The selection of the inbred lines was performed to avoid low fertility and aberrant morphology. Therefore, one of the reasons for the observed high heterozygosity may have been the existence of loci associated with inbreeding depression (Zhang et al., 2019). Further analyses are necessary to investigate this hypothesis.

Because the objective of this study was to predict the F_1_ hybrid phenotype in strawberry, we incorporated the dominant effect in trait heritability estimation and GS model construction. We then compared the results with the models to those with the additive effect only. In both heritability and GS model accuracy, additive plus dominant effect models showed higher values than additive effect models in most cases (Tables 1, 2). The advantage of additive plus dominant effect models was not statistically significant in GS model accuracy (Table 2). As reported in previous studies, large, extensive datasets are necessary for statistical testing to determine the advantage of dominant effects. Therefore, the inclusion of dominant effects in GS models has resulted in insignificant improvements in most studies (Varona et al., 2018). Nevertheless, the use of additive plus dominant effect models in this study showed a slight advantage in GS accuracy, but did not show a disadvantage against additive only effect models (Table 2). Moreover, the comparison of GS predicted values between some F_1_ hybrids and their parents indicated that there were over-dominant effects in some parental combinations (Supplementary Figure 3). These results suggest that the inclusion of dominant effects in the GS model is preferable for hybrid breeding, even if the advantage is not clear in cross-validations.

In the across population prediction, GS accuracy of Brix was lower than that of the other traits (Table 3), despite the fact that the GS accuracy seemed reasonable in within-population cross-validation (Table 2). The low accuracy in Brix may be attributable to genotype-by-environment interaction effects due to differences in year of phenotyping between the populations (Figure 1B). In most GS studies, phenotype data were obtained over replication for several years. However, in projects at the initial stage, such as strawberry F_1_ hybrid breeding, such data are not available; therefore, data-driven approaches are necessary. Nevertheless, our results indicated that phenotype data obtained from long-term replication are necessary to avoid the risk of overestimating GS accuracy due to unexpected genotype-by-environment interaction effects.

The most important advantage of GS is that it enables breeding selection without phenotypic observation. This means not only breeding selection at the seedling stage, but also the selection of parental combinations for better progenies (Iwata et al., 2013; Yamamoto et al., 2017). The latter matches the situation of hybrid breeding in this study. Therefore, we conducted a pilot experiment for GS in strawberry F_1_ hybrid breeding. For this objective, we developed 21 F_1_ hybrids that consisted of five classes with different characteristics (Figure 4A). The phenotypic values of the F_1_ hybrids were well correlated with the GS predicted values, indicating that GS is applicable for strawberry hybrid breeding (Figures 4B,C). In this study, we focused on fruit hardness and pericarp color because these are important targets of our breeding project (Table 1). The most preferable characteristics in our breeding project are both high fruit hardness and high pericarp color. Unfortunately, we could not find such F_1_ hybrids not only in the observed phenotypic values, but also in the GS predicted values (Figure 4A). To obtain F_1_ hybrids that have both high fruit hardness and high pericarp color traits, other breeding strategies such as recurrent selection may be necessary (Yamamoto et al., 2016).

## Data Availability Statement

The datasets and R scripts presented in this study can be found in https://github.com/yame-repos/FxaF1GS. The sequence data from the WGS libraries are available in the DDBJ Sequence Read Archive (SRA) under accession number DRA011267.

## Author Contributions

EY conducted modeling of genomic selection, while SK conducted phenotyping and breeding selection based on genomic selection models. KS and SI conducted genotyping of the materials in this study. SK and YN constructed the 105 inbred lines and the 257 test F_1_ hybrids. EY and SK wrote the manuscript. All authors contributed to the article and approved the submitted version.

## Conflict of Interest

The authors declare that the research was conducted in the absence of any commercial or financial relationships that could be construed as a potential conflict of interest.

## Footnotes

1 http://faostat3.fao.org

2 http://hannonlab.cshl.edu/fastx_toolkit/index.html

3 http://strawberry-garden.kazusa.or.jp/

## Supplementary Material

Supplementary Figure 1 Distribution of phenotypic values. The upper and lower panels indicate the 105 inbred lines and the 275 test F_1_ hybrids, respectively. **(A)** Petiole length; **(B)** leaf area; **(C)** brix; **(D)** fruit hardness; and **(E)** pericarp color.

Supplementary Figure 2 Frequency of heterozygous genotypes in the SNPs detected. The vertical red line indicates the threshold (0.25) used for filtration (see section “Materials and Methods”).

Supplementary Figure 3 Comparison of GS predicted values between the F_1_ hybrids and their parents. The predicted values were calculated using Genomic best linear unbiased prediction (GBLUP) with an additive plus dominant effect model. P1 and P2 on the *x*-axes indicate the parents of the F_1_ hybrids. F_1_ hybrids and their parents are connected with solid lines. The black and white circles indicate the F_1_ hybrids selected for high- and low-pericarp color, respectively. The black and white squares indicate the F_1_ hybrids selected for high- and low-fruit hardness, respectively. The black triangles indicate the F_1_ hybrids selected for intermediate phenotypes. **(A)** Petiole length; **(B)** leaf area; **(C)** brix; **(D)** fruit hardness; and **(E)** pericarp color.
