# Supplementary figures and images for: Genomic Selection for F1 Hybrid Breeding in Strawberry (Fragaria × ananassa)
Source: Front Plant Sci. 2021 Mar 4;12:645111. doi: 10.3389/fpls.2021.645111 (PMC7969887; doi:10.3389/fpls.2021.645111)

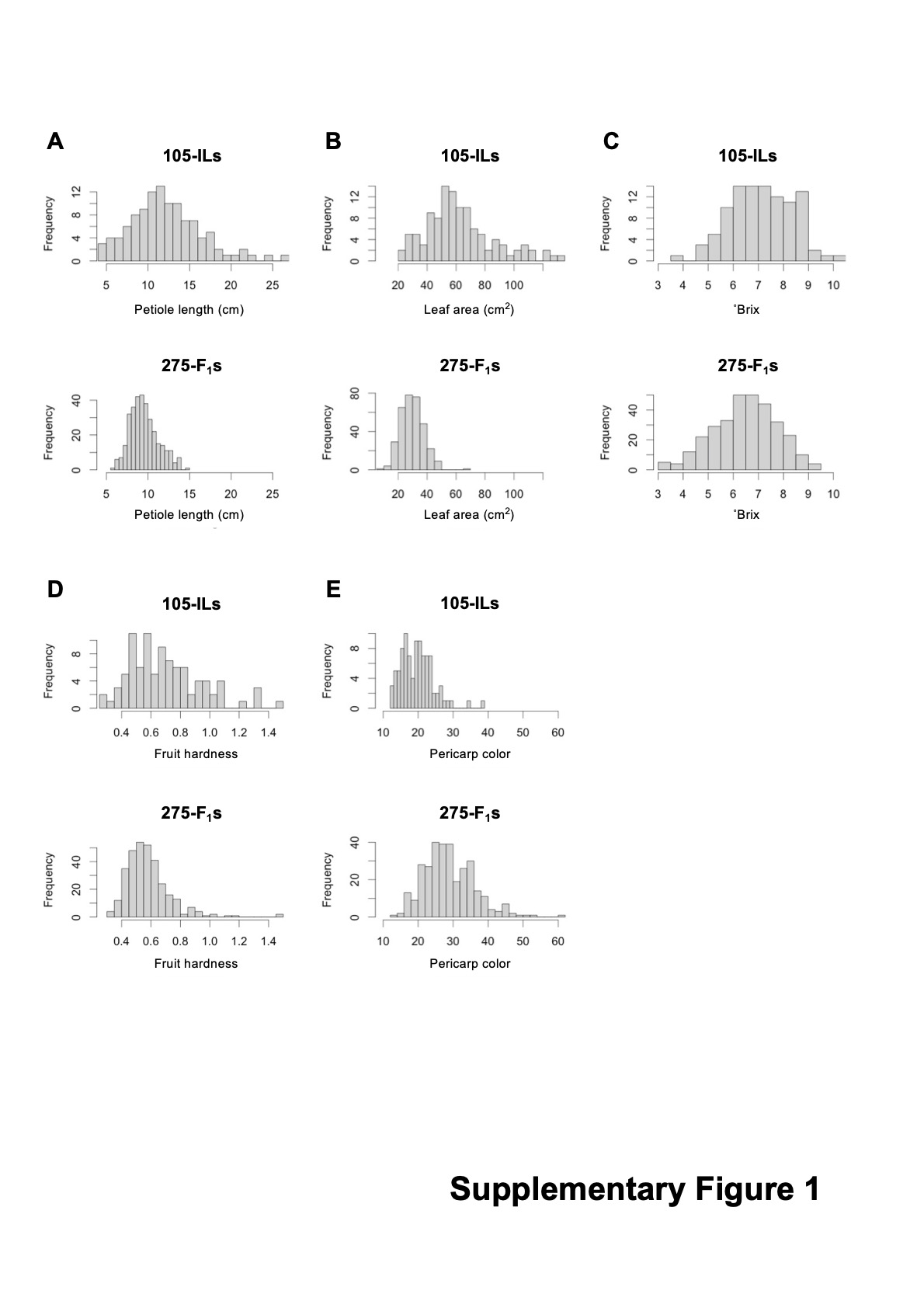

Supplement: Supplementary Figure 1 — Distribution of phenotypic values. The upper and lower panels indicate the 105 inbred lines and the 275 test F1 hybrids, respectively. (A) Petiole length; (B) leaf area; (C) brix; (D) fruit hardness; and (E) pericarp color. [file Image_1.JPEG]

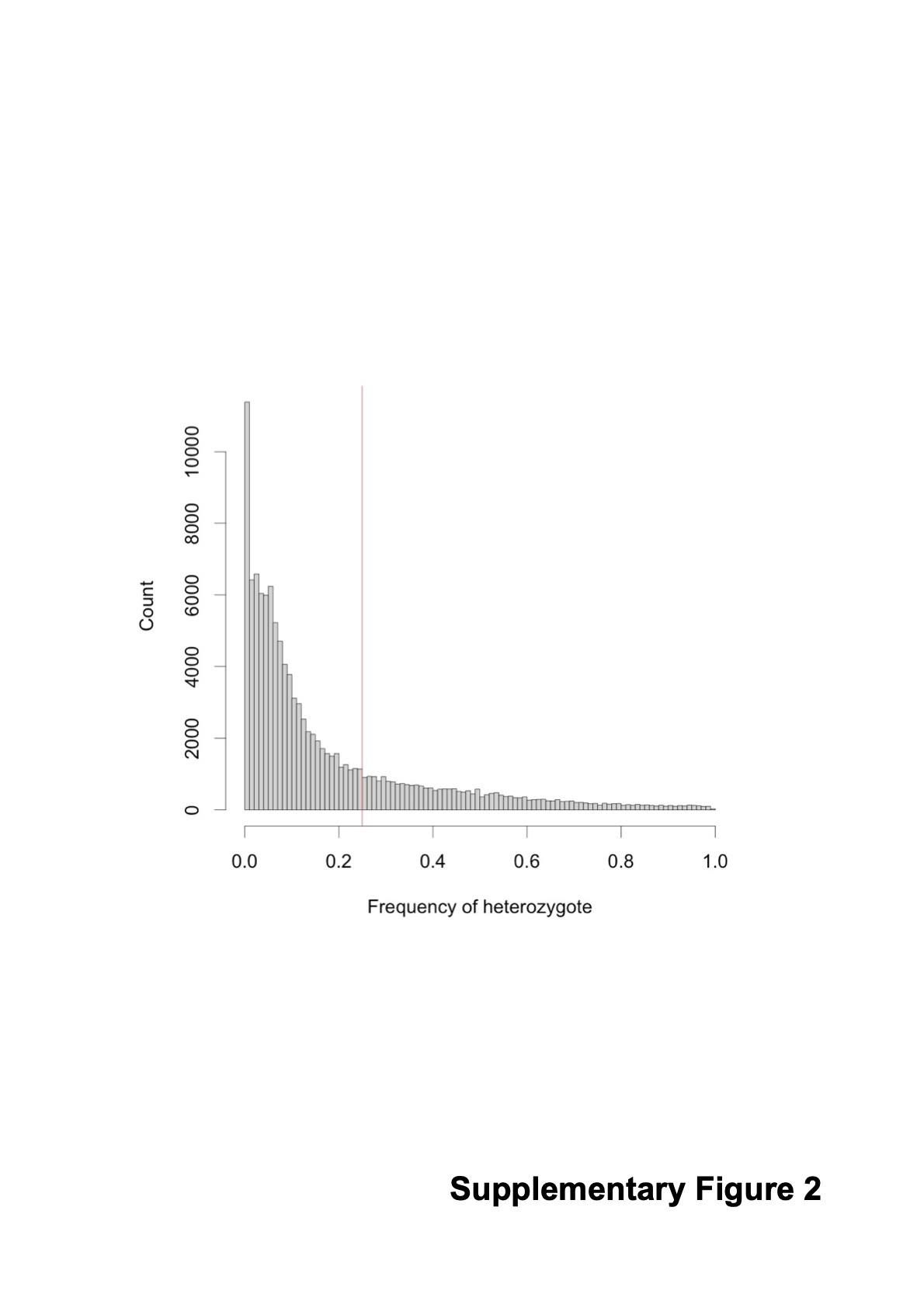

Supplement: Supplementary Figure 2 — Frequency of heterozygous genotypes in the SNPs detected. The vertical red line indicates the threshold (0.25) used for filtration (see section “Materials and Methods”). [file Image_2.JPEG]

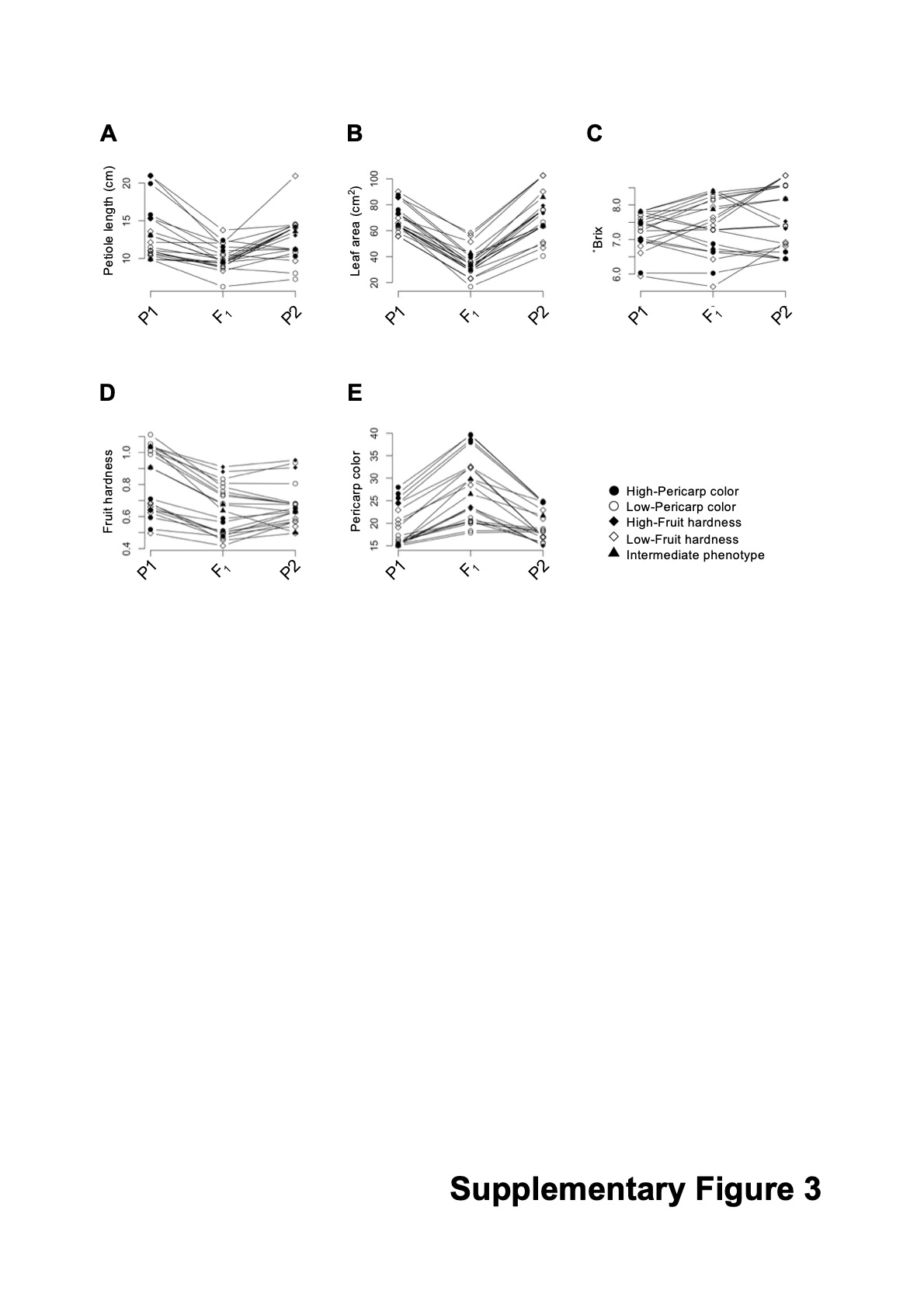

Supplement: Supplementary Figure 3 — Comparison of GS predicted values between the F1 hybrids and their parents. The predicted values were calculated using Genomic best linear unbiased prediction (GBLUP) with an additive plus dominant effect model. P1 and P2 on the x-axes indicate the parents of the F1 hybrids. F1 hybrids and their parents are connected with solid lines. The black and white circles indicate the F1 hybrids selected for high- and low-pericarp color, respectively. The black and white squares indicate the F1 hybrids selected for high- and low-fruit hardness, respectively. The black triangles indicate the F1 hybrids selected for intermediate phenotypes. (A) Petiole length; (B) leaf area; (C) brix; (D) fruit hardness; and (E) pericarp color. [file Image_3.JPEG]
